# Unraveling the unphosphorylated STAT3–unphosphorylated NF-κB pathway in loss of function STAT3 Hyper IgE syndrome

**DOI:** 10.3389/fimmu.2024.1332817

**Published:** 2024-08-20

**Authors:** Adil Karim, Rashi Garg, Biman Saikia, Abha Tiwari, Smrity Sahu, Mehak Malhotra, Ranjana W. Minz, Amit Rawat, Surjit Singh, Deepti Suri

**Affiliations:** ^1^ Department of Immunopathology, Postgraduate Institute of Medical Education and Research, Chandigarh, India; ^2^ Department of Pediatrics, Postgraduate Institute of Medical Education and Research, Chandigarh, India

**Keywords:** inborn error of immunity (IEI), primary immunodeficencies (PID), Hyper IgE syndrome (HIES), signal transducer and activator of transcription 3 (STAT3), Regulated upon Activation, normal T-cell expressed and secreted chemokines (RANTES), nuclear factor kappa b (NFκB)

## Abstract

**Background:**

Patients with loss of function signal transducer and activator of transcription 3-related Hyper IgE Syndrome (LOF STAT3 HIES) present with recurrent staphylococcal skin and pulmonary infections along with the elevated serum IgE levels, eczematous rashes, and skeletal and facial abnormalities. Defective STAT3 signaling results in reduced Th17 cells and an impaired IL-17/IL-22 response primarily due to a compromised canonical Janus kinase-signal transducer and activator of transcription (JAK–STAT) pathway that involves STAT3 phosphorylation, dimerization, nuclear translocation, and gene transcription. The non-canonical pathway involving unphosphorylated STAT3 and its role in disease pathogenesis, however, is unexplored in HIES.

**Objective:**

This study aims to elucidate the role of unphosphorylated STAT3–unphosphorylated NF-κB (uSTAT3–uNF-κB) activation pathway in LOF STAT3 HIES patients.

**Methodology:**

The mRNA expression of downstream molecules of unphosphorylated STAT3–unphosphorylated NF-κB pathway was studied in five LOF STAT3 HIES patients and transfected STAT3 mutants post-IL-6 stimulation. Immunoprecipitation assays were performed to assess the binding of STAT3 and NF-κB to RANTES promoter.

**Results:**

A reduced expression of the downstream signaling molecules of the uSTAT3–uNF-κB complex pathway, viz., *RANTES*, *STAT3*, *IL-6*, *IL-8*, *ICAM1*, *IL-8*, *ZFP36L2*, *CSF1*, *MRAS*, and *SOCS3*, in LOF STAT3 HIES patients as well as the different STAT3 mutant plasmids was observed. Immunoprecipitation studies showed a reduced interaction of STAT3 and NF-κB to RANTES in HIES patients.

**Conclusion:**

The reduced expression of downstream signaling molecules, specially *RANTES* and *STAT3*, confirmed the impaired uSTAT3–uNF-κB pathway in STAT3 LOF HIES. Decreased levels of RANTES and STAT3 could be a significant component in the disease pathogenesis of Hyper IgE Syndrome.

## Graphical Abstract

Impaired downstream signaling cascade of unphosphorylated STAT3–unphosphorylated NF-κB. (uSTAT3–uNF-κB) pathway in the pathogenesis of loss of function STAT3 Hyper IgE syndrome (LOF STAT3 HIES).

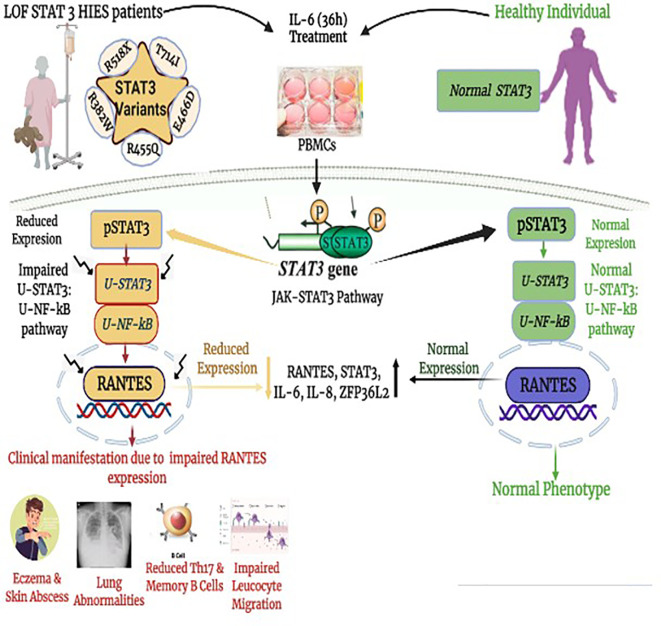

## Introduction

Loss of function STAT3 Hyper IgE syndrome (LOF STAT3 HIES) is a rare inborn error of immunity characterized by a clinical triad of high serum IgE (>2,000 IU/mL), skin abscesses, and pneumonia (1). The clinical manifestations of Hyper IgE syndrome (HIES) include both immunological and non-immunological abnormalities. The immunological features include elevated serum IgE, eosinophilia, reduced neutrophil chemotaxis, *Staphylococcus* skin infections, and pneumonia along with reduced Th17 cells, memory B cells, and STAT3 phosphorylation. The non-immunological features include facial dysmorphism (prominent forehead, retained primary teeth, and increased nasal width) and skeletal abnormalities (scoliosis and hyperextensibility) (2–8). Various cytokines, mainly IL-6 and IL-23, that initiate STAT3 phosphorylation on tyrosine 705 residue lead to homo- or hetero-dimer formation through Src Homology 2 (SH2) domain interactions (9, 10). The resulting STAT3 dimerization leads to its nuclear translocation that finally acts as a transcription factor of various target genes like *RANTES*, *STAT3*, *IL-6*, *IL-8*, *ICAM1*, *IL-8*, *ZFP36L2*, *CSF1*, *MRAS*, and *SOCS3*, the latter being a negative regulator of STAT3 (11, 12).

STAT3 and NF-κB interaction is known (13), and it has been shown that when cells are stimulated with IL-1 and IL-6, STAT3 forms a complex with the p65 subunit of NF-κB and the bound STAT3 interacts with the non-consensus sequence close to the κB elements of the serum amyloid A (SAA) promoter. Furthermore, it has been demonstrated that a complex that includes STAT3, NF-κB p65, and p300 is required for the synergistic activation of the SAA gene in response to IL-1 and IL-6 (14). STAT3 and NF-κB p65 physically interact *in vivo*, and NF-κB p65 homodimers may collaborate with unphosphorylated STAT3 (uSTAT3) when linked to κB motifs (15). In addition, the NF-κB p50 subunit can collaborate with phosphorylated STAT3 (pSTAT3) that is bound to the gamma activating sequence (GAS) of DNA (15).

STAT3 has been shown to play a vital role in facilitating gene expression without tyrosine 705 (Y705) phosphorylation as an uSTAT3. uSTAT3 binds to unphosphorylated NF-κB (uNF-κB) in response to IL-6 that results in the uSTAT3–uNF-κB complex formation that accumulates in the nucleus and further act as a novel transcription factor (16). This unique mechanism of uSTAT3 in mediating gene expression is completely distinct from classical (canonical) phosphorylated STAT3 (pSTAT3). STAT3 is regulated by its own activation because its promoter, similar to STAT1, contains GAS elements that drive its own expression in response to the activation of STAT3. Long-term treatment to IL-6 leads to an increase in the concentration of uSTAT3, and hence the levels of uSTAT3 are dependent on STAT3 phosphorylation (17, 18). The role of this uSTAT3–uNF-κB pathway is, however, unexplored in patients with LOF STAT3 HIES. With this background, we tried to elucidate the role of the uSTAT3–uNF-κB complex activation pathway in the pathogenesis of LOF STAT3 HIES patients by analyzing the downstream signaling molecules of the uSTAT3–uNF-κB pathway in response to IL-6. Our data showed an impaired activation of the uSTAT3–uNF-κB pathway that resulted in the downregulation of *RANTES* and *STAT3* and other κB-dependent genes like *IL-6*, *IL-8*, *ICAM1*, *IL-8*, *ZFP36L2*, *CSF1*, *SOCS3*, and *IFNB1* in LOF STAT3 HIES patients.

The role of chemokines in regulating the immune response in STAT3 HIES is largely unexplored. We studied the significance of Regulated on Activation, Normal T Expressed, and Secreted (RANTES) in HIES patients and looked for a correlation with their clinical findings. RANTES is highly expressed during chronic infections compared to acute infections (19, 20). LOF STAT3 HIES patients have normal myeloid and lymphoid cell development, but they have impaired antigen-dependent differentiation and function (21–25). Our study demonstrated the significantly reduced expression of RANTES in two out of five patients. However, three patients showed a near-normal expression of RANTES. RANTES is a crucial molecule in the uSTAT3–uNF-κB complex pathway (16, 26), and the reduced levels of RANTES in two of our HIES patients correlated with more severe clinical manifestations. Transfection studies with different STAT3 mutants further confirmed the impaired activation of the uSTAT3–uNF-κB pathway in a STAT3-deficient state.

## Results

### Five families of LOF STAT3 HIES with nonsense or missense variants in STAT3

**Figure 1:**
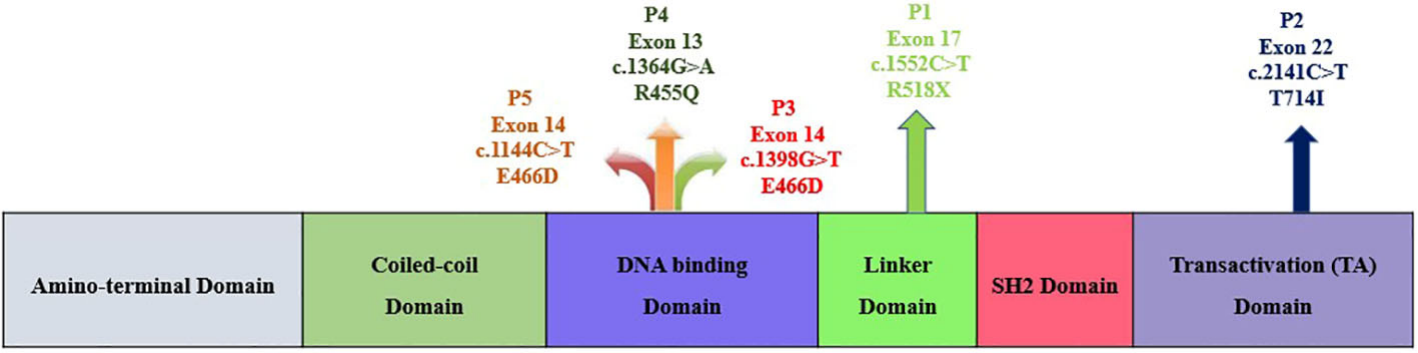
Schematic diagram depicting the five study subjects with LOF STAT3 HIES harboring either nonsense or missense variants within the STAT3 gene.

This study was carried out in the Department of Immunopathology, Postgraduate Institute of Medical Education and Research (PGIMER), Chandigarh, India, from 2017 to 2022. Five LOF STAT3 HIES patients with pathogenic variants in different domains of STAT3 gene, viz., linker domain (P1; R518X), transactivator domain (P2; T714I), and DNA binding domain (P3; E466D, P4; R455Q and P5; R382W), were enrolled in the study (**Figure 1**). The patients were scored according to the HIES scoring system (NIH Score) using the categories of 0–15 as unaffected, 16–39 as possible, 40–59 as probable, and ≥60 as definitive HIES. Patients who had a NIH score of ≥20, high serum IgE levels (>1,500 IU/mL), and reduced Th17 levels less than 0.5% were included in this study (**Table 1**). As per the American College of Medical Genetics and Genomics (ACMG) guidelines, all variants examined in our study were categorized either as pathogenic (*n* = 3) or likely pathogenic (*n* = 2). A total of 10 healthy controls (HCs) without any history of illness and with normal serum IgE levels were recruited in this study. The study protocol was approved by the Institute Ethics Committee (IEC-03/2018-870), and all patients were recruited after obtaining a written informed consent/assent. The clinical photographs and clinical manifestation of five recruited LOF STAT3 HIES patients are mentioned in **Supplementary Figure 1**.

**Table 1:** Demographic data: clinical, immune cell population and mutation details of the five recruited HIES patients.

S. No	Age/Sex	IgE Levels (IU/ml)	NIH Score	pSTAT3	tSTAT3	TH17 Cells (%)	Memory B cells	Mutation	Domian	Amino Acid Change	ACMG Classification	SIFT Score
P1	2.5Y/M	9752	46	45.80%	66.90%	0.30%	4.20%	Exon 17 c.1552C>T	Linker	R518X	Pathogenic	0
P2	3Y/M	4300	53	6.50%	58.50%	0.20%	3.10%	Exon 22 c.2141C>T	Transactivator	T714I	Pathogenic	0
P3	34Y/F	1869	15	35.80%	93.60%	0.40%	7.10%	Exon 14 c.1398G>T	DNA Binding	E466D	Likely Pathogenic	0.01
P4	6Y/M	5200	22	23.30%	70.40%	0.50%	16.60%	Exon 13 c.1364G>A	DNA Binding	R455Q	Likely Pathogenic	0
P5	3Y/F	2135	29	19.50%	65.90%	0.10%	2.20%	Exon 13 c.1144C>T	DNA Binding	R382W	Pathogenic	0

NIH Score, A clinical scoring system was devised by the National Institutes of Health (NIH) group who recognized  LOF STAT3-HIES to be a multisystem disorder.

ACMG, The ACMG (American College of Medical Genetics and Genomics) guidelines indicated that all the variants were classified as either pathogenic (n=3) or likely pathogenic (n=2).

SIFT Score: The variant's SIFT Score (Sorting Intolerant from Tolerant) is zero, indicating an impact on protein function. A score ranging from 0 to 0.05 is predicted to affect protein function.

### Reduced expression of phosphorylated STAT3, total STAT3, and Th17 and Th1 cells in LOF STAT3 HIES patients

**Figure 2:**
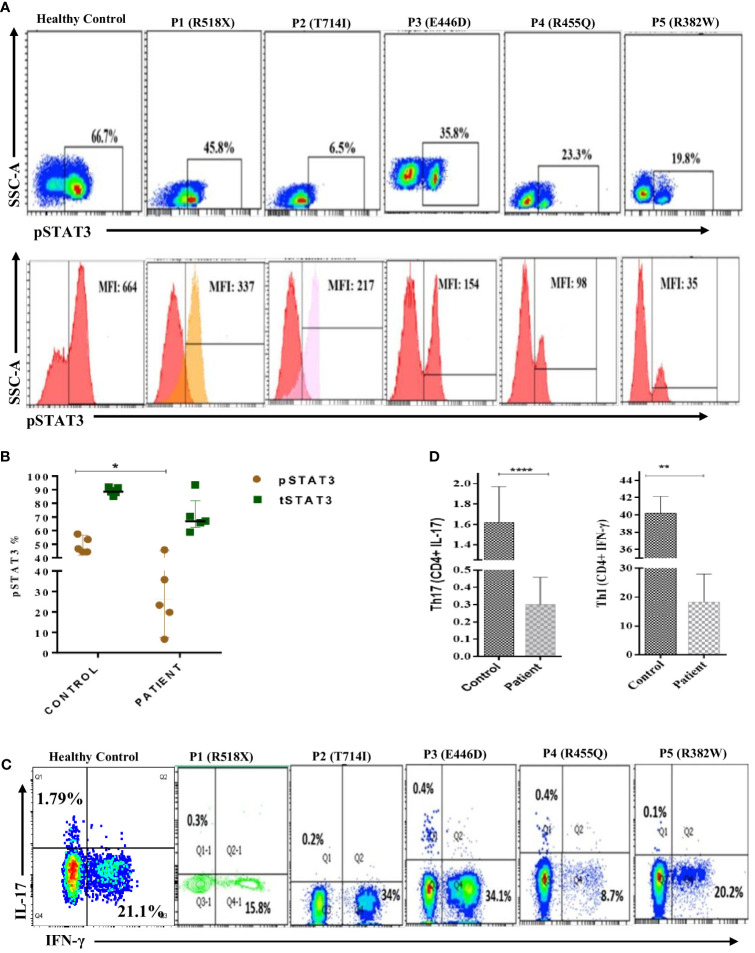
Representative flow cytometry contour plots and histograms of phosphorylated-STAT3 (pSTAT3) in **(A)** control in comparison with five patients. Unstimulated histogram peaks are on the left side and stimulated peaks are on the right side of each individual plot. **(B)** Scatter plot showing pSTAT3 and total STAT3 (tSTAT3) distribution in patients and healthy controls (HCs) after IL-6 stimulation. pSTAT3 is significantly decreased (p < 0.05*) in patients compared to HCs, whereas tSTAT3 is comparable, not significant (ns). **(C)** Contour density plot showing the reduced expression of IL-17A and IFN-g in CD4+ cells in patients compared to HCs. **(D)** Bar graph showing the significantly reduced expression of Th17 (*p* < 0.0001****) and Th1 (*p* < 0.01**) cells in patients compared to HCs. ANOVA test was used to compare the healthy control group with the five patients. Graphs show mean ± SD.*P<0.05, **P < 0.01, ****P<0.0001, NS-not significant.

Flow cytometry analysis revealed a significant reduction (*p* < 0.05) in phosphorylated STAT3 (pSTAT3) levels after IL-6 stimulation in four out of five patients (**Figures 2A, B**). Meanwhile, the expression of total STAT3 (tSTAT3) remained comparable between patients and healthy controls (**Supplementary Figure 2**). In the healthy control group, the range of pSTAT3 spans from 44.4% to 57.5%, while tSTAT3 ranges from 84% to 92% across all five HCs. The percentage of CD4+ IL-17A+ cells (Th17), assessed after stimulation with phorbol 12-myristate 13-acetate (PMA) and ionomycin, exhibited a significant reduction in all five HIES patients, ranging from 0.1% to 0.4% (**Table 1**). Additionally, the range of Th17 in the control group varies from 1.1% to 1.9%. CD4+ IFNγ+ cells (Th1) were also significantly reduced in all patients, except patient 2, compared to the healthy controls (**Figures 2C, D**).

### Differential gene expression of downstream signaling molecules in the uSTAT3–uNF-κB pathway in LOF STAT3 HIES

**Figure 3:**
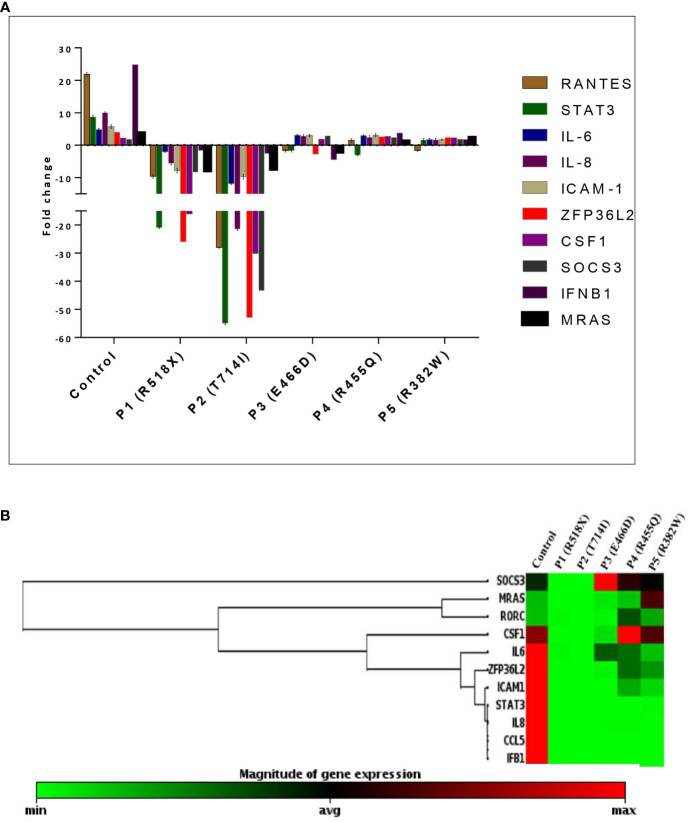
**(A)** Bar graph and mean fold change expression of selected genes in healthy controls (HCs) *versus* LOF STAT3 HIES patients when stimulated with IL-6 (160 ng/mL) for 36 h. The primary transcript for each gene was assayed at least in triplicate and normalized to β-actin and GAPDH. **(B)** Clustergram of differential gene expression in LOF STAT3 HIES patients (*n* = 5) and healthy controls (*n* = 10). The color ranges from green to red through black, according to the magnitude of relative gene expression. Targets are clustered according to their similarity in the expression pattern. ANOVA test was performed to compare the healthy control group with the five HIES patients. A *p*-value of ≤0.05 was considered to be significant.

To assess the downstream signaling molecules of the uSTAT3–uNF-κB complex pathway, a comparison of gene expression was performed in cultured peripheral blood mononuclear cells (PBMCs) derived from HIES patients and HCs after IL-6 stimulation for 36 h. The relative mRNA expression of *RANTES* [fold change (FC); 21.89 ± 0.58], *STAT3* (FC; 8.69 ± 0.57), *IL-6* (FC; 4.84 ± 0.39), *IL-8* (FC; 10.6 ± 0.21), *ICAM-1* (FC; 5.79 ± 0.58), *ZPF36L2* (FC; 4.02 ± 0.26), *CSF1* (FC; 2.17 ± 0.51), *SOCS3* (FC; 1.91 ± 0.60), *IFNβ1* (FC; 24.87 ± 0.88), and *MRAS* (FC; 3.76 ± 0.21) was found to be upregulated in HCs after IL-6 treatment. On the other hand, patients 1 and 2 showed a downregulation of all genes, whereas patient 3 showed a comparative downregulation of *RANTES* (FC; -1.68 ± 0.43), *STAT3* (FC; -1.65 ± 0.46) *IL-6* (FC; 3.08 ± 0.21), *IL-8* (FC; 2.82 ± 0.38), *ICAM-1* (FC; 2.99 ± 0.33), *ZPF36L2* (FC; -2.79 ± 0.92), *CSF1* (FC; 1.82 ± 0.70), *SOCS3* (FC; 2.97 ± 0.33), *IFNβ1* (FC; -4.49 ± 0.69), and *MRAS* (FC; -1.71 ± 0.21) compared to the HCs. However, patients 4 and 5 showed up to twofold upregulation of the studied genes except *RANTES* (-1.5 ± 0.54) and STAT3 (-3.04 ± 0.21) as well as *RANTES* (-1.64 ± 0.32) and STAT3 (-1.54 ± 0.49) for patients 4 and 5, respectively, but the fold change was still found to be significantly lower than those of HCs (**Figure 3**).

### Reduced binding of STAT3–NF-kB p50 to RANTES promoter in LOF STAT3 HIES

**Figure 4:**
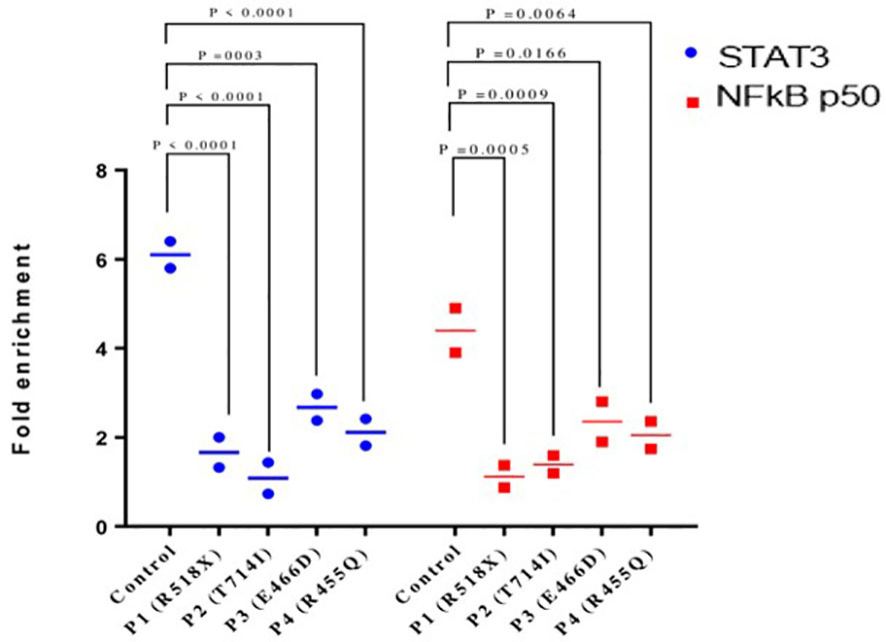
Chromatin immunoprecipitation qPCR (ChIP qPCR) analysis for STAT3 and NF-kB p50 binding to RANTES promoter after treatment with IL-6 (160 ng/mL) for 36 h. Quantification of fold enrichment in healthy control and HIES patients relative to nonspecific IgG as a negative control and normalized with input DNA was done. ANOVA test was utilized to compare the healthy control group with the five HIES patients. A *p*-value of ≤0.05 was considered to be significant.

Chromatin immunoprecipitation (ChIP) qPCR assay was performed using anti-STAT3 antibody followed by anti-NF-κB p50 antibody to confirm that both proteins bind simultaneously to the RANTES promoter. The ChIP qPCR data showed an increase of STAT3 and NF-κB p50 binding to the RANTES promoter with 6.1- and 4.4-fold upregulation, respectively, compared to non-specific IgG in HCs after treatment with IL-6. On the other hand, STAT3 and NF-κB p50 binding to the RANTES promoter was significantly decreased in all four patients. The fold enrichment of STAT3 and NF-κB p50 in the patients was 1.6- and 1.1-fold in patient 1, 1.08- and 1.3-fold each in patient 2, and 2.6- and 2.3-fold each in patients 3 and 4, respectively (**Figure 4**). These findings indicate the compromised binding of STAT3 and NF-κB p50 to the RANTES promoter in patients with HIES, and this compromised binding is attributed to the reduced pSTAT3 levels, which are acting as its own regulator.

### Reduced STAT3 protein expression in patients in response to IL-6 compared to HCs

**Figure 5:**
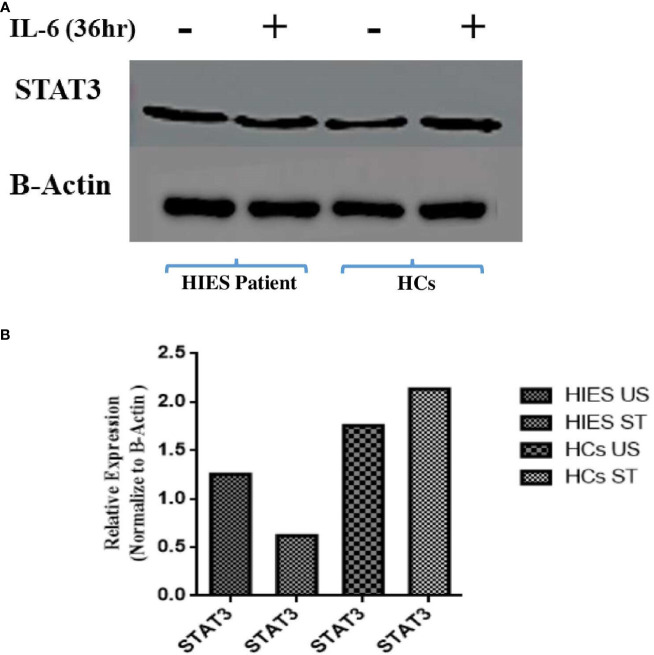
**(A)** Western blot analyses assessing STAT3 protein expression in response to IL-6 stimulation (160 ng/mL) for 36 h in both healthy controls and HIES subject. The depicted images serve as representatives illustrating the variations in STAT3 protein levels in response to the IL-6 stimulus. Representative images showing the expression of STAT3 and NF-kB p50. **(B)** Densitometry was done using image J software using β-actin. Unpaired *t*-test was used to assess comparisons between HIES subjects in the unstimulated and stimulated conditions.

To elucidate the effects of long-term IL-6 stimulation on STAT3 protein levels in HIES patients, PBMCs from patients and HCs were subjected to stimulation with IL-6 (160 ng/mL), followed by Western blot analysis which revealed a distinct pattern. In the IL-6-stimulated HIES PBMCs, we observed a reduction in STAT3 protein levels compared to their unstimulated counterparts. Conversely, in the HCs, we observed an opposite trend, where IL-6 stimulation led to an increase in STAT3 protein expression compared to the unstimulated samples (**Figure 5**).

### Effect of STAT3 mutant plasmids on the signaling molecules of the uSTAT3–uNF-κB pathway

**Figure 6:**
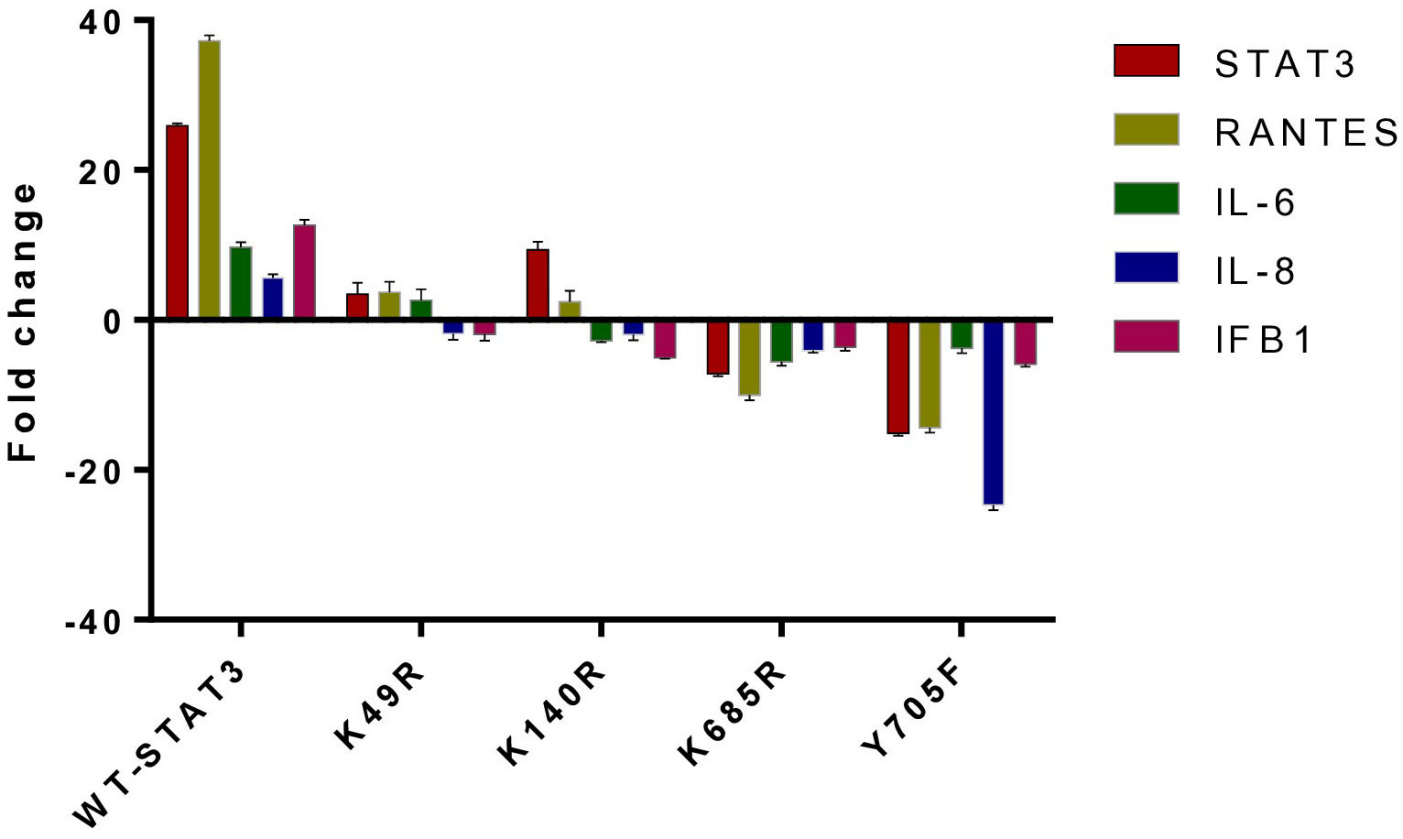
The relative gene expression of STAT3, RANTES, IL-6, IL-8, and IFN-β1 in Jurkat cells transfected with various STAT3 mutants was analyzed following a 36-h treatment with interleukin-6 (IL-6) at a concentration of 160 ng/mL. A bar graph illustrating the mean fold change expression of each gene in different STAT3 mutants compared to WT-STAT3 when stimulated with IL-6 for 36 h is presented. An ANOVA test was conducted to compare the wild-type STAT3 (WT-STAT3) with all five STAT3 mutants. A *p*-value of ≤0.05 was considered to be significant.

To examine the effect of different STAT3 mutants involving different domains on the downstream signaling molecules of the uSTAT3–uNF-kB pathway in response to IL- 6, we procured pLEGFP plasmids of four different STAT3 mutants (Y705F STAT3, K49R STAT3, K140R STAT3, and K685R STAT3) with a FLAG tag (**Supplementary Figure 3**). The relative gene expression of *STAT3*, *RANTES*, *IL-6*, *IL-8*, and *IFN-β1* in STAT3 mutant transfected in Jurkat cells was assessed by qRT-PCR after treatment with IL-6 for 36 h. In cells expressing wild-type STAT3 (WT-STAT3), a mean fold change of 25.9 ± 0.28 in *STAT3*, 37.25 ± 0.70 in *RANTES*, 9.690 ± 070 in *IL-6*, 5.63 ± 0.43 in *IL-8*, and 12.36 ± 0.70 in *IFNβ1* was observed. Different transfected STAT3 mutants showed a differential fold change expression compared to WT-STAT3 (**Figure 6** and **Table 2**). The amino terminal domain (AT) and coiled-coil domain (CC) mutants K49R and K140R, respectively, were able to specially upregulate the mRNA expression of *RANTES* and *STAT3*, while SH2 and TA domain mutants K685R and Y705F showed a reduced expression of all genes when compared to WT-STAT3. The fold changes of each gene in transfected cells compared to WT-STAT3 are depicted in **Table 2**.

**Table 2:** Fold change expression of genes in STAT3 mutants: mean fold change of gene profiles in WT-STAT3 and transfected STAT3 mutants when stimulated with IL-6 (160 ng/mL) for 36 h. [A negative sign (&ndash;) indicates downregulated expression.].

Genes	WT-STAT3 Vs K49R-STAT3 (Fold change)	WT-STAT3 Vs K140R-STAT3 (Fold change)	WT-STAT3 Vs K685R-STAT3 (Fold change)	WT-STAT3 Vs Y705F-STAT3 (Fold change)
***STAT3***	3.40±1.2	9.335 ±1.082	-7.170 ±0.354	-15.130±0.283
***RANTES***	3.67±1.457	2.45±1.478	-10.02 ±0.707	-14.38 ±0.636
***IL-6***	2.660 ±1.471	-2.770 ±0.184	-5.540 ±0.566	-3.750 ±0.707
***IL-8***	-1.86 ±0.764	-1.95 ±0.771	-4.070 ±0.283	-24.7 ±0.636
***IFNβ1***	-1.910 ±0.834	-5.020 ±0.141	-3.640 ±0.481	-5.910 ±0.354

### Reduced expression of signaling molecules of uSTAT3–uNF-κB in HIES patients and correlation with their disease phenotype

**Table 3** shows the comparative analysis of the expression of downstream signaling molecules of the uSTAT3–uNF-κB pathway, which were then correlated with the severity of the clinical manifestations in the recruited HIES patients, viz., recurrent infections, lung anomalies, and facial abnormalities (prominent forehead, retained primary teeth, and increased nasal width). The NIH score ranges from 15 to 53 in patients. A higher NIH score more accurately predicts the severity of HIES patients. A Combined Annotation-Dependent Depletion (CADD) score is a numerical measure used in bioinformatics to assess the deleteriousness of genetic variants. CADD predicts a continuous phred-like score that ranges from 1 to 60, with higher values indicating more deleterious cases (27). All patients have a high CADD score that ranged from 28 to 49.

**Table 3:** Comparative analysis of the clinical manifestations of LOF STAT3 HIES patients (n=5), immune cell populations, and cytokine gene expressions.

	Patient 1	Patient 2	Patient 3	Patient 4	Patient 5
**Age/Sex**	2.5Y/M	3Y/M	34 Y/F	6 Y/M	3Y/F
**Mutation**	Exon 17 c.1552C>T	Exon 22 c.2141C>T	Exon 14 c.1398G>T	Exon 13 c.1364G>A	Exon 13 c.1144C>T
**NIH Score**	46	53	15	22	29
**CADD Score**	40	31	28.9	33	32
**IgE levels**	9752	4300	1869	5200	2135
**Eosinophilia**	>800	>800	<700	<700	<700
**Eczema**	Moderate	Moderate	Mild	Mild	Mild
**Immunological Features**	Low IgG Normal IgA & IgM	Normal IgG, IgA & IgM	Normal IgG, IgA & IgM	Low IgG, IgM & Normal IgA	Variable IgG, Normal IgA , IgM
**Immunoglobulin**					
**Th17 cells**	0.3% (Reduced)	0.2% (Reduced)	0.4% (Reduced)	0.5% (Reduced)	0.1% (Reduced)
**Th1 cells**	15.8% (Reduced)	34.00%	12.5% (Reduced)	8.7% (Reduced)	20.2% (Reduced)
**pSTAT3**	45.80%	6.50%	35.80%	23.30%	19.50%
**Total STAT3**	66.90%	58.50%	93.60%	70.40%	65.90%
**Memory B cells**	4.20%	3.10%	7.10%	16.60%	2.20%
**Infections**	Severe	Severe	Mild	Mild	Mild
**Skin Abscess**					
**Pneumonia**	<2	<3	None	2 Episodes	3 Episodes
**(Over Lifetime)**					
**Candidiasis**	Oral	Oral	None	None	None
**Viral Infections**	Present	Present	None	Absent	Absent
**Newborn Rash**	Present	Present	Absent	Absent	Absent
**Differential cytokines expression**	Reduced	Reduced	Normal	Normal	Normal
***RANTES***					
***STAT3***	Reduced	Reduced	Reduced	Reduced	Normal
***IL-6***	Reduced	Reduced	Normal	Normal	Normal
***IFβ1***	Reduced	Reduced	Normal	Normal	Normal
***CSF1***	Reduced	Reduced	Normal	Normal	Normal
**Lung Anomalies**	Present	Present	Absent	Absent	Absent
**Bronchiectasis**					
**Pneumatocele**	Absent	Present	Absent	Absent	Absent
**Facial Abnormalities**	Present	Present	Absent	Mildly Present	Absent
**Prominent forehead**					
**Cathedral palate**	Present	Present	Absent	Absent	Absent
**Retained Primary Teeth**	2	2	None	None	1
**Increased Nasal Width**	1-2 SD	1-2 SD	<1 SD	<1 SD	<1 SD

CADD, Combined Annotation-Dependent Depletion (CADD) score is a numerical measure used in bioinformatics to assess the deleteriousness of genetic variants.

CADD predicts a continuous phred-like score that ranges from 1 to 60, higher values indicating more deleterious cases19. All they have high CADD score that ranged from 28 to 49.

The clinical features were prominent, especially in patients 1 and 2. All patients had reduced CD4+IL17A+ cells (Th17) with a range of 0.1% to 0.5%. Reduced CD4+IFN-γ+ cells (Th1) were observed in the majority of patients (four out of five) that ranged from 8.5% to 20.1%, except in P2 (T714I) who had a normal expression of CD4+IFN-γ+ cells (34.0%). On analyzing whether the expression levels of the downstream signaling molecules of this pathway, viz., RANTES, STAT3, IL-6, CSF1, and IFNβ correlated with their clinical severity, the following observations were made (**Table 3**): P1 and 2 had normal levels of CD4+ T helper cells (45.1% and 39.5%, respectively), but they had reduced levels of *RANTES*, *STAT3*, *IL-6*, *CSF1*, and *IFNβ* expression. In contrast to P3, patients 4 and 5 had an almost normal expression of *RANTES*, *IL-6*, *CSF1*, and *IFNβ.* However, patients 3, 4, and 5 had a normal expression of *RANTES* compared to P1 and P2, and they did not have severe infection and lung anomalies.

## Discussion

STAT3 is a critical component of the JAK–STAT signaling pathway that controls the expression of numerous genes that are involved in various cellular processes, including cell survival, differentiation, proliferation, inflammation, apoptosis, and oncogenesis (3, 28–30). STAT3 gene mutation was identified to be the cause of the autosomal dominant form of Hyper IgE Syndrome (AD-HIES) (31–34) that results in impaired Th17 cell differentiation (35–38) and increased susceptibility to bacterial and fungal infections, especially *Candidiasis* (39). All five LOF STAT HIES subjects in our study had a significantly reduced number of Th17 (CD4+IL-17A+) and Th1 (CD4+IFNγ+) cells, except in patient 2. The intricate interplay between Th17 and Th1 cell differentiation involves shared signaling pathways and transcription factors. Consequently, a mutation affecting STAT3 may perturb the delicate balance between Th17 and Th1 cells, thereby impacting the expression of both T cell subsets (40–42).

It has been demonstrated that high levels of STAT3 lead to the constitutive activation of many genes like *RANTES*, *MRAS*, *CCND1*, and *CDC2*, which are involved in various biological processes and are essential in cell cycle and oncogenesis (26, 43, 44).

In our investigation into the impact of STAT3 deficiency on the gene expression profiles, we noted the dysregulation of certain genes in LOF STAT3 HIES patients, particularly in those with mutations affecting the transactivation (TA) and linker domains, which significantly impair STAT3 phosphorylation. Conversely, mutations in the DNA-binding domain (DBD), known for retaining normal STAT3 phosphorylation, exhibited a relatively preserved gene expression compared to healthy controls (HCs).

For instance, patient 1 carried a nonsense mutation (R518X) in the linker domain, resulting in a truncated STAT3 protein lacking the SH2 and transactivation domains. In contrast, patient 2 had a mutation (T714I) in the TA domain near the tyrosine phosphorylation site. Patient 1 exhibited an absent SH2 domain, while patient 2 displayed reduced STAT3 phosphorylation. This led to a decreased accumulation of uSTAT3 in the cytoplasm, hampering its interaction with unphosphorylated NF-κB (uNF-κB) and compromising the formation of the uSTAT3–uNF-κB complex. Consequently, this impaired complex formation resulted in reduced binding to the promoter site of RANTES and the subsequent downregulation of genes involved in this pathway, including SOCS3.

In normal circumstances, activated STAT3 induces the expression of negative regulators like SOCS3 to curtail excessive immune activation. However, in the context of reduced STAT3 phosphorylation, this feedback loop may not be adequately triggered, leading to the downregulation of SOCS3. This downregulation was more pronounced in patients 1 and 2 with mutations in the linker and SH2 domains, respectively, compared to patients 4 and 5 with mutations in the DBD. These three patients (3, 4, and 5) therefore had normal STAT3 phosphorylation, and as a result, the downstream signaling genes of the uSTAT3–uNF-κB pathway were relatively preserved, including SOCS3. Thus, elucidating the differential impact of STAT3 mutations on gene expression profiles sheds light on the intricate mechanisms underlying the pathophysiology of HIES.

Our ChIP qPCR data showed an increased interaction of STAT3 and NF-κB p50 to the RANTES promoter in the HCs after IL-6 stimulation. This interaction would be expected to induce another wave of gene expression that is mediated by the unphosphorylated state of STAT3 and NF-κB and acts as novel transcriptional factors which are distinct from the phosphorylated STAT3 (16). However, due to reduced STAT3 phosphorylation in HIES patients, this STAT3 and NF-κB p50 interaction to the RANTES promoter would be compromised that it will lead to a reduced expression of downstream signaling molecules.

The STAT3 mutant plasmids employed in this study are documented to be loss of function (LOF) in nature (45). Our results showed different gene expression patterns for the different STAT3 transfected mutants. Amino terminal (AT) domain mutants are able to transcribe genes after IL-6 stimulation as they were able to bind to the DNA binding domain (45, 46). In the same study, K685R mutant was found to have a minor impact on IL-6-mediated STAT3 gene regulation, while Y705F mutant was found to abolish SOSC3 transcription and STAT3 nuclear transportation (46–48). Hence, the varying effects of distinct domain HIES STAT3 variants observed in our study correspond with earlier studies on the mutant plasmids (45, 46). We observed SH2 (K685R) and TA (Y705F) domain mutants to have a diminished expression of all major downstream signaling molecules of the uSTAT3–uNF-κB pathway. However, N-terminal (K49R) and CC (K140R) domain mutants were able to transcribe genes, but to a lesser extent in response to IL-6. The major downstream signaling molecules of the uSTAT3–uNF-κB pathway are *RANTES* and *STAT3*, and distinct gene expression patterns of both *RANTES* and *STAT3* were observed for each mutant. The SH2 and TA domain mutants showed a significant downregulation of *RANTES* and *STAT3*; however, N-terminal and CC domain mutants could transcribe *RANTES* and *STAT3*, albeit to a lesser extent. These patterns of gene expression of the *STAT3* mutants were very similar to that of our HIES patients.

The role of chemokines in regulating the immune response in LOF STAT3 HIES is largely unexplored. RANTES is highly expressed during chronic infections compared to acute infections (19, 20, 49, 50) and is also a key mediator for acute and chronic inflammation in various other immunopathological disorders (51–54). The reduced expression of RANTES also results in defective CD8 T-cell migration, suggesting that RANTES has an important role in regulating the optimal immune response during infections (20, 49, 55, 56). In response to IL-6, RANTES induces the tyrosine phosphorylation of STAT3 that leads to an increase in unphosphorylated STAT3 (43). This consequently triggers a subset of κB-activated genes, among which RANTES is included. The kB element of the RANTES promoter could work in two ways: directly in response to TNF-α or IL-1 or indirectly in response to IL-6 (43). In our study, we observed a reduced RANTES expression in two out of five patients, while three patients exhibited nearly normal RANTES expression levels.

The decreased RANTES expression in patients 1 and 2 was associated with more severe clinical symptoms, possibly due to compromised leukocyte recruitment or function. This led to more pronounced clinical features such as skin abscesses, recurrent infections, and lung anomalies (bronchiectasis). Conversely, patients 3, 4, and 5 had an almost normal RANTES expression and did not display severe clinical manifestations compared to patients 1 and 2. RANTES is a potent chemoattractant mainly involved in recruitment (51, 52), which targets a wide range of leukocytes, including memory T cells, eosinophils, and monocytes. Depending on the cellular environment, RANTES can help to deliver the chemoattractant or deliver activating signals that induce dendritic cells for the production of cytokines (57, 58).

The disrupted uSTAT3–uNF-κB axis observed in STAT3 LOF HIES, leading to a diminished RANTES response, likely plays a significant role in the disease’s pathogenesis. Although this study did not encompass patients with IL-6 and IL-6ST defects, a comparable mechanism is anticipated. This research introduces a novel perspective on disease pathogenesis, potentially carrying therapeutic implications and offering a potential disease marker.

## Methods

### DNA extraction and mutational analysis

Genomic DNA (gDNA) was isolated by using QiAmp kit according to the manufacturer’s protocol. A full-length STAT3 gene was amplified and sequenced from gDNA covering exons 2 to 3 (N-terminal domain), exons 3–9 (CC domain), exons 10–16 (DBD), exons 16–19 (Linker domain), exons 19–21b (SH2 domain), and exons 21–23 (TA domain) (**Figure 1**). The amplified products were outsourced for Sanger sequencing, and mutation analysis of the sequenced samples was done using Codon Code Aligner software (Codon Code Corporation, USA).

The clinical scoring system developed by the National Institutes of Health (NIH) includes the NIH clinical feature score as well as the determination of IL-17-producing T cells (59). The ACMG guidelines (60) were used for the interpretation of genetic variants. A variant’s SIFT (Sorting Intolerant from Tolerant) score ranging from 0 to 0.05 is predicted to affect protein function (61).

### PBMC culture

A total of 1 × 10^6^ PBMCs were cultured in RPMI 1640, treated with IL-6 (Peprotech, USA), and maintained in a humidified atmosphere in a CO_2_ incubator. After optimization, PBMCs were incubated either as unstimulated samples or stimulated with IL-6 for 36 h (160 ng/mL).

### Intracellular staining of phosphorylated STAT3, total STAT3, and IL-17 and IFN-γ

A total of 1 × 10^6^ PBMCs from HIES subjects and HCs were stimulated with IL-6 for 36 h (160 ng/mL), permeabilized by using chilled methanol, and subsequently incubated for 30 min at room temperature and further stained with Alexa flour 647-labeled anti-pSTAT3 (Y705) and PE-labeled anti-STAT3 antibody (BD Biosciences, USA). The pSTAT3 (Y705) antibody recognizes the phosphorylated tyrosine 705 position of STAT3, while the anti-STAT3 antibody recognizes Stat3 (isoform 1) regardless of the phosphorylation status. For Th1 and Th17 analysis, cells were stimulated for 4–6 h with 10 ng/mL PMA, 1 ug/mL ionomycin, and 10 μg/mL Brefeldin A and further stained with PE-labeled anti-CD4 antibody (BD Biosciences, USA). Permeabilization was done using 200 μL of BD Cytofix buffer and incubation for 30 min at 4°C. Staining was done with Alexa flour 647-labeled anti-IL17A and FITC-labeled anti-IFN-γ (BD Biosciences, USA) for 30 min at room temperature. The stained cells were then washed and acquired on BD LSR Fortessa (BD Biosciences, USA) and analyzed by FACS Diva 8.0 software.

### RNA extraction, cDNA synthesis, and customized PCR array

Total RNA was isolated from Trizol (Ambion, Life Technologies, USA), according to the TRI Reagent protocol (Merck, Sigma). cDNA synthesis was carried out using Revert Aid™ First-strand cDNA Synthesis Kit (Fermentas Life Sciences, USA) according to the manufacturer’s instructions. PCR arrays were customized by Qiagen Molecular Diagnosis, India. Relative gene expression was performed in cultured PBMCs from HIES patients (*n* = 5) and HCs (*n* = 10) along with Jurkat cells transfected with STAT3 mutant plasmids after treatment with IL-6 for 36 h. Briefly, cDNA was mixed with SYBR Green PCR master mix. Equal volumes of this mixture were added to each well of the 96-well PCR array plate containing the pre-dispensed gene-specific primer sets in duplicate, and qPCR was performed by using Roche LC 480 thermal cycler (Roche Diagnostic, Germany). The details of the amplification program used are as follows: initial denaturation at 95°C for 10 min, followed by 45 cycles of denaturation at 95°C for 15 s, annealing at 60°C for 10s, and finally extension at 72°C for 20 s. Finally, the fold change expression of each gene was calculated using RT^2^ PCR Array analysis software by applying the 2^−(ΔCt)^ formula.

### Chromatin immunoprecipitation

Chromatin immunoprecipitation (ChIP) assays were performed according to the manufacturer’s protocol (CST ChIP Kit cat. no. 9005). DNA was extracted from both a HIES patient and HC PBMCs using the manufacturer’s protocol (CST, USA, cat. #10010), and purified DNA was used as a template for PCR with specific primers. The primers for ChIP-qPCR were designed from primer BLAST tool, and the sequences were as follows: *RANTES* forward primer 5´-GCAAGTCACTCCTGCTCACT-3´ and *RANTES* reverse primer 5´-GCCAGCCACTATTCCACTGT-3´. Both input and immune-precipitated DNA were quantified by real-time PCR with the above-mentioned primers.

### Western blotting

Protein lysates from a LOF STAT3 HIES patient and healthy control were quantified using BCA assay, and SDS-PAGE was conducted by loading protein samples at concentrations ranging from 30 to 50 μg/mL. After electrophoresis, the gels were washed with double-distilled water and subsequently prepared for western blotting. The proteins separated through SDS-PAGE were then transferred onto a nitrocellulose membrane, and blocking was carried out using 4% bovine serum at 4°C overnight. Following the blocking process, the membrane was washed three times with 0.2% TBST, and anti-STAT3 antibody (cat. #4904 CST, USA) was applied at 1:2,000 dilution. After the incubation period, the membranes were rinsed three times with TBST for 5 min each. Subsequently, the appropriate anti-rabbit IgG HRP-linked antibody (at 1:3,000 dilution) was added, and this was left on a shaker for 2 h at room temperature. Finally, the ECL substrate was added to the membrane following the kit’s instructions, and the reactive bands were visualized using the chemiluminescence system (FlourChem E, USA).

### Transfection of STAT3 mutants in Jurkat cells

Jurkat cell line was procured from the National Centre for Cell Science, Pune, India. The cells were maintained in RPMI 1640, supplemented with 10% FBS and antibiotics. The cells were grown as monolayers at 37°C in 5% CO_2_ in a six-well plate until 60%–70% confluence and were then suspended in RPMI 1640 and antibiotic cocktail for at least half an hour before transfection. Lipofectamine complex was prepared using 1 µg of plasmid and 0.75 µL of Lipofectamine reagent 3000 and 2 µL of Lipofectamine (Thermofisher Scientific). The cells were treated for 6 h with the complex, following which complete media was added for 48 h. The cells were observed for transfection efficiency using Alexa flour 647-tagged anti-FLAG antibody (cat. #2368S, Cell Signaling Technologies, USA) by flow cytometry.

### Statistical analysis

Statistical analysis was performed using GraphPad Prism software, version 5.0 (GraphPad Software, USA). Comparisons between groups of related samples were assessed by paired or unpaired *t*-test. Mann–Whitney test was performed for non-parametric data. ANOVA test was performed for more than two group comparisons. A *p*-value ≤0.05 was considered to be significant.

### Clinical implications

The activation of the uSTAT3–uNF-κB pathway might contribute significantly to the disease phenotype observed in individuals with LOF STAT3 HIES. Additionally, RANTES may also serve as an important biomarker for this particular patient group.

## Data availability statement

The original contributions presented in the study are included in the article/**Supplementary Materials**, further inquiries can be directed to the corresponding author/s.

## Ethics statement

The studies involving humans were approved by Institutional Ethics Committee, Postgraduate Institute of Medical Education and Research (PGIMER), Chandigarh. The studies were conducted in accordance with the local legislation and institutional requirements. Written informed consent for participation in this study was provided by the participants’ legal guardians/next of kin. Written informed consent was obtained from the individual(s), and minor(s)’ legal guardian/next of kin for the publication of any potentially identifiable images or data included in this article.

## Author contributions

AK: Conceptualization, Data curation, Formal analysis, Funding acquisition, Methodology, Resources, Software, Validation, Visualization, Writing – original draft, Writing – review & editing. RG: Formal analysis, Methodology, Writing – review & editing. BS: Conceptualization, Funding acquisition, Investigation, Project administration, Supervision, Validation, Visualization, Writing – review & editing. AT: Methodology, Writing – review & editing. SSa: Methodology, Writing – review & editing. MM: Formal analysis, Writing – review & editing. RM: Investigation, Supervision, Writing – review & editing. AR: Supervision, Writing – review & editing. SSi: Supervision, Writing – review & editing. DS: Supervision, Writing – review & editing.
