# Supplementary material for: Unraveling the unphosphorylated STAT3–unphosphorylated NF-κB pathway in loss of function STAT3 Hyper IgE syndrome
Source: Front Immunol. 2024 Aug 20;15:1332817. doi: 10.3389/fimmu.2024.1332817 (PMC11369709; doi:10.3389/fimmu.2024.1332817)
Supplement: Supplementary file 1 [file DataSheet_1.pdf]

Supplementary figures:

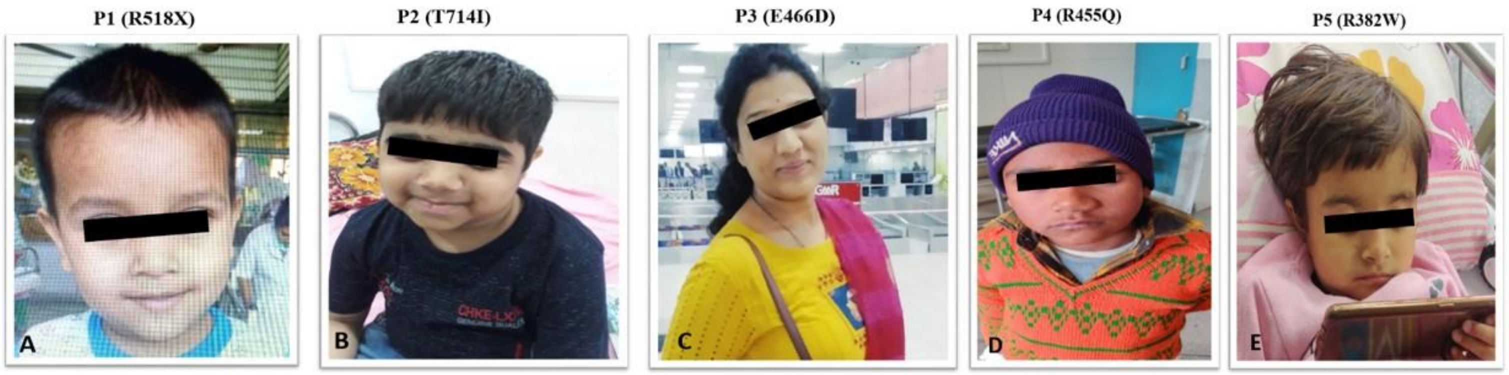

Supplementary Fig 1: Clinical manifestation of five recruited HIES subjects **A)** Facial appearance of Case 1 (2/M) with prominent forehead and broad nose. **B)** Case 2 (5/M) showing coarsening of facies along with prominent forehead & broad nose. **C)** Case 3 (34/F) had complained of an itchy lesion on the right side of the ear. She had no facial manifestation. **D)** Case 4 (6/M) shows mild facial features, including a broad forehead, deep-set eyes & bulbous nose. **E)** Facial appearance of Case 5 (2/F) with a prominent forehead and broad nasal inter-canthal distance

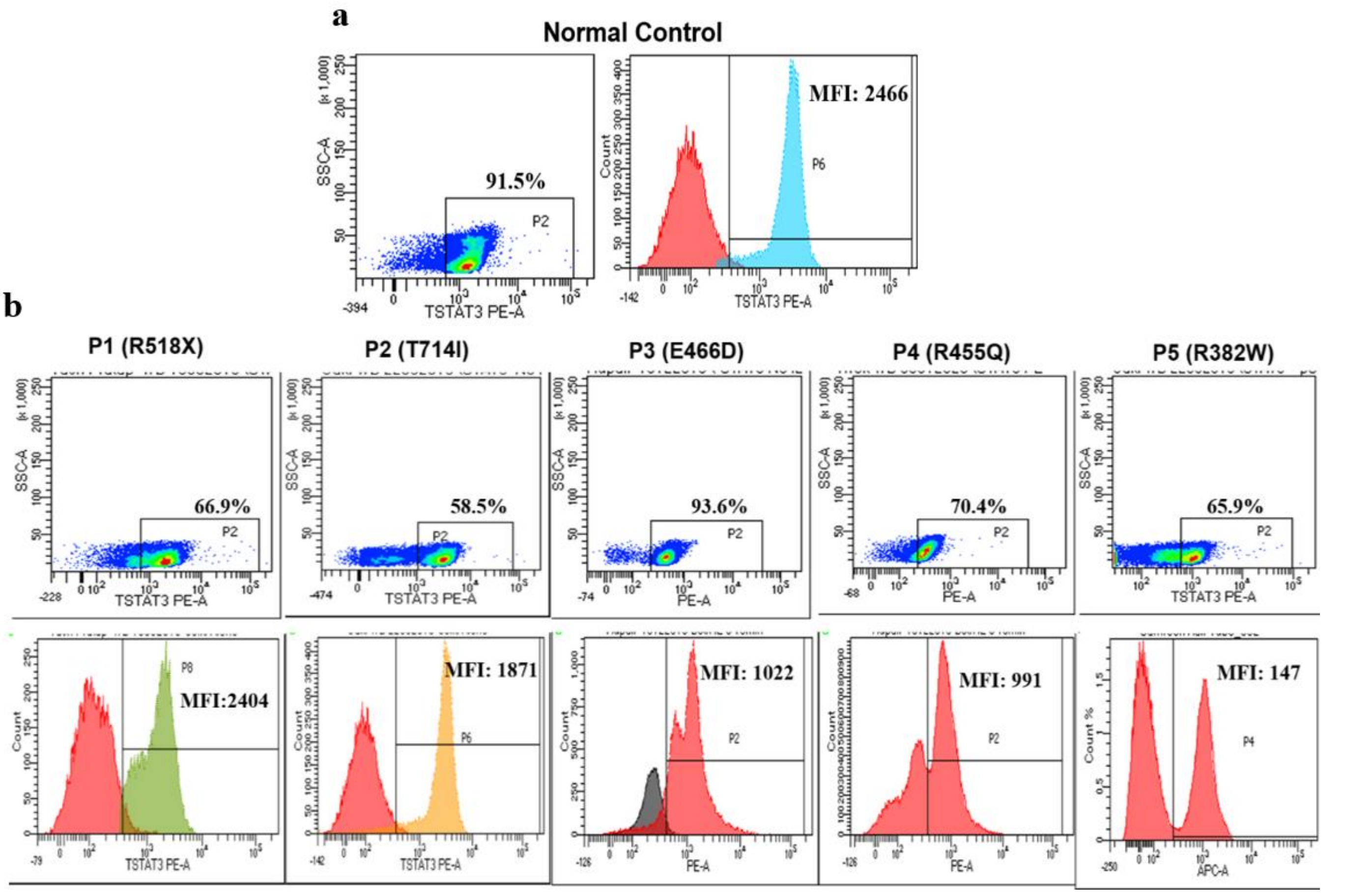

Supplementary Figure 2: Representative contour plots & histogram of flow cytometry analysis of total STAT3 (tSTAT3) in normal control **a)** in comparison with LOF STAT3 HIES patients **b)**. Unstimulated histogram peaks were on the left side and stimulated peaks on the right side of each individuals plots. Percentages and MFI of cells positive for tSTAT3 are detailed within the histogram boxes.

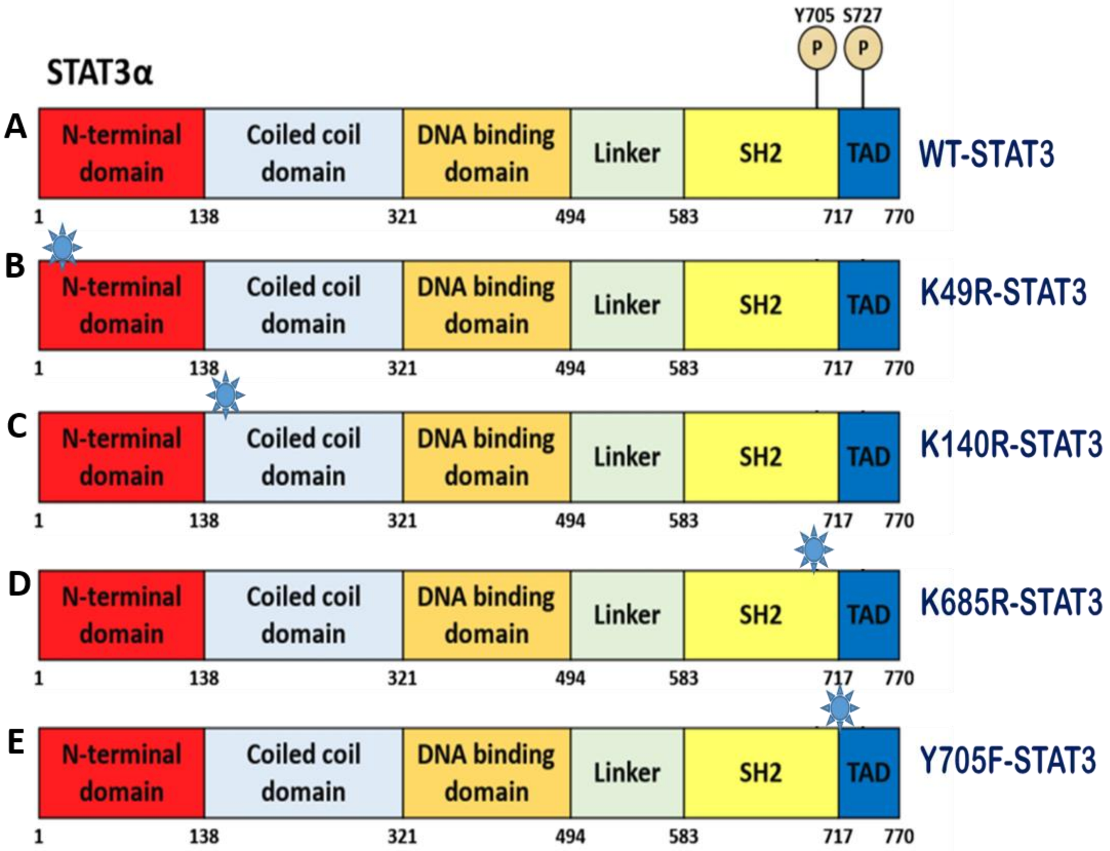

Supplementary Figure 3: Schematic representation of STAT3 structure in pLEGFP plasmid: Structure **A** shows the WT -STAT3, **B** represents the N-terminal domain STAT3 mutant with K49R mutation. Structure **C** and **D** gives a representation of K140R, Coiled-coil domain STAT3 mutant and K685R SH2 domain mutant respectively, while structure **E** represents TA domain STAT3 mutant with Y705F. (adapted from Lim et al, 2005).
